# Folding by Numbers: Primary Sequence Statistics and Their Use in Studying Protein Folding

**DOI:** 10.3390/ijms10041567

**Published:** 2009-04-08

**Authors:** Brent Wathen, Zongchao Jia

**Affiliations:** Department of Biochemistry, Queen’s University, Kingston, Ontario, Canada K7L 3N6

**Keywords:** Primary Sequence, Protein Folding, Sequence-Structure Relationship

## Abstract

The exponential growth over the past several decades in the quantity of both primary sequence data available and the number of protein structures determined has provided a wealth of information describing the relationship between protein primary sequence and tertiary structure. This growing repository of data has served as a prime source for statistical analysis, where underlying relationships between patterns of amino acids and protein structure can be uncovered. Here, we survey the main statistical approaches that have been used for identifying patterns within protein sequences, and discuss sequence pattern research as it relates to both secondary and tertiary protein structure. Limitations to statistical analyses are discussed, and a context for their role within the field of protein folding is given. We conclude by describing a novel statistical study of residue patterning in *β*-strands, which finds that hydrophobic *(i,i+2)* pairing in *β*-strands occurs more often than expected at locations near strand termini. Interpretations involving *β*-sheet nucleation and growth are discussed.

## 1. Introduction

It has been almost five decades since Anfinsen and colleagues first confirmed that the folding instructions for ribonuclease A are contained in its primary amino acid sequence [1]. This landmark finding has led to a tremendous amount of research into what has become known as the protein folding problem [2–5], an apt encapsulation for a body of theoretical, biochemical and computational work that aims to unravel the relationship between a protein’s primary sequence and its resulting three-dimensional structure. In addition to answering fundamental questions about the basic machinery of life – a satisfying enterprise in its own right – a solution to this challenging problem promises to open up tremendous new frontiers in medicine and protein engineering.

Despite substantial theoretical [6–8], experimental [9–11], and computational [12–14] advances, the means by which an unfolded polypeptide chain achieves its final biological conformation continues to evade detailed characterization [13]. Experimentally, the folding process is extremely challenging to study. Important parts of this process, particularly the earliest stages in folding, happen on time scales that are not accessible to almost all biochemical techniques (though there has been considerable progress on this front [15]). In addition, the protein folding data that is available typically pertains to the thermodynamics and kinetics of a whole population of molecules, each following its own particular folding trajectory, with little information available about the specific folding mechanisms themselves [16]. What can be determined from thermodynamic and kinetic results, moreover, suggests that the folding process contains numerous small energy barriers with few – and quite often no – isolatable intermediates [17]. To a large extent, then, the folding process remains a mechanistic “black box” phenomenon, with few specifics established with certainty.

Theoretically, much of the progress in protein folding over the past couple of decades has come from the use of statistical mechanics. Though it has its detractors [18], the Landscape Model of protein folding – which contends that proteins have funneled energy landscapes with many routes down their folding funnels towards their native conformations – has much support within the folding community [7,8,15,19–21]. Unfortunately, the theory has yet to foster a breakthrough in protein structure prediction. Of the significant computational hurdles to successful prediction, the most notable one is simply the sheer magnitude of the conformational search. Captured in what is known as Levinthal’s paradox, the conformational search that a folding protein must perform to find its native structure, even with substantial simplifications, would take longer than the age of the universe were it done randomly; that most proteins fold on timescales measured in seconds is taken as proof that the folding process cannot be random, but rather must follow some sort of pathway [3]. Yet without the knowledge of folding pathways or even the basic rules of folding, computational methods are often reduced to searching for a needle in an enormous haystack. The most successful approaches to date have made very good use of known protein structures to predict unknown ones, but on the whole, much of their success reflects expertise in copying and not in folding *per se* [22].

While the protein folding process continues to evade researchers, examples of the end result of this process abound. Indeed, the fact that the protein folding problem remains unsolved has not limited structural biochemists, who have continued to determine protein structures at a staggering pace. As this rich repository of structural information grows, it is natural to wonder whether it can be used to unravel, if not the entire folding process, then at least parts of it, through the identification of statistically significant patterns. It is not surprising, then, that the structural data has been subjected to intense scrutiny for many years, including several attempts to redefine the structural building blocks of proteins [23–25], in the hope of uncovering keys to the relationship between protein sequence and protein structure. Here, we provide an introduction into the use of statistics for finding and analyzing patterns in protein sequences within the context of the protein folding problem. As the sheer magnitude of this topic cannot be covered in detail here, our aim is to focus on the elementary types of statistical research that have been done on protein sequences, and, through highlighting the conclusions and interpretations that have been drawn from this research, provide some idea of the power of these basic techniques. Specifically, this survey presents the use of statistics for studying *(i)* protein primary sequences; *(ii)* the relationship between protein sequence and secondary structure; and *(iii)* connections between protein sequence and tertiary structure. Throughout, we discuss the strengths of this approach for studying protein folding, as well as its limitations. As a final example, we close with a re-examination of residue patterning within *β*-strands that demonstrates that the type of analysis one performs can directly affect the nature of the results one obtains.

## 2. Statistical Analyses of Protein Primary Sequences

We begin by looking at research that has examined the statistical nature of residue patterning in protein sequences without regards to protein structure. Our focus is on studies of residue patterning, and not on statistical methods for comparing sequences, such as would be found in sequence alignment work. For a recent review of sequence alignment, see [26].

### 2.1. The Randomness of the Primary Sequence

The study of protein sequence data has a long history [27]. One of the earliest analyses of a large ensemble of protein sequences was done by White and Jacobs, who examined the distribution of hydrophobic residues along protein chains [28]. Hydrophobicity has long been considered to be a main driving force behind protein folding [6,29,30], and so one would anticipate that the distribution of hydrophobic residues within protein chains is central to protein folding. By tabulating the frequencies of hydrophobic runs, however, White and Jacobs found that the distribution of hydrophobic residues within protein chains cannot readily be distinguished from random [28]. This study is noteworthy for a number of reasons, not the least of which being the surprising nature of its findings. From a practical point of view, this work demonstrates the use of a very frequently employed simplification in statistical studies: alphabet reduction. Instead of focusing on patterns involving all twenty amino acids, researchers can choose to cluster amino acids together into groupings based on shared physiochemical properties or other features of interest such as homolog substitution frequency [31], local structural environments [32], or tertiary structural environments [33]. In this case, White and Jacobs grouped residues by water affinity into hydrophobic and polar classes. This particular alphabet reduction is used extensively throughout the literature [34], though there is considerable discrepancy with regards to amino acid class assignments, complicating cross-study generalizations. The conclusions from this study are also of particular interest, as they illustrate the scope of statistical implications: White and Jacobs conclude that the random nature of the distribution of hydrophobic residues in proteins chains suggests *(i)* that functional proteins may have developed from purely random sequences, and *(ii)* that protein folding may be more permissive with regards to amino acid sequence specificity than is generally thought [28]. This study provides a clear example of how a mix of statistics and deduction can be used to investigate a wide range of protein phenomena, from their distant origins to properties about their folding processes.

The nature of the distribution of hydrophobic residues within proteins has been examined by other researchers, who have drawn different conclusions. Vazquez *et al.*, for example, as well as King and co-workers, concluded after examining such distributions that longer runs of hydrophobic residues occur significantly less often than expected [35–37]. The contrast between these conclusions and those of White and Jacobs demonstrate an important characteristic of the use of statistics: different methodologies can lead to different results. For example, Vazquez *et al.* suggest that their results oppose those of White and Jacobs because of differences between their sets of hydrophobic residues: (VLIFM) in the case of Vazquez *et al.* [35] as compared to (VLIFMAPWYCG) in the study by White and Jacobs [28]. King and co-workers, on the other hand, have chosen a hydrophobic classification (VLIFMAPWY) [36,37] that is closer to that of White and Jacobs, and so the discrepancy between the major findings of these two studies is unlikely to be the result of different alphabet reduction. Instead, these authors attribute this discrepancy to a subtle difference in calculation methods: in their work, they examine the hydrophobic distributions across their database as a whole, whereas White and Jacobs examine hydrophobic distributions within protein sequences individually [28].

### 2.2. Binary Patterns Within Primary Sequences

Other statistical studies of protein sequences have used a different approach, examining the frequencies of binary patterns along protein chains instead of residue distributions. Vazquez *et al.*, in the same study cited above, looked at the frequency of all possible five-residue binary patterns in their protein dataset, where the binary alphabet was again composed of hydrophobic and polar residues [35]. Those patterns that contained pairs of adjacent hydrophobic residues surrounded by polar ones, or those with hydrophobic residues separated by two polar ones, had frequencies that were statistically higher than expected, while patterns with alternating hydrophobic and polar residues were found to be significantly suppressed. Vazquez *et al.* cite these findings as further evidence for a non-random distribution of hydrophobic residues within proteins. Moreover, they draw an interesting inference about these types of statistical approaches to the study of protein folding: examinations of patterning within protein sequences without regard to secondary structure may provide insights into folding events prior to secondary structure formation, including the ultra-early events in protein folding. Specifically, they suggest that favoured sequence patterns may have been selected by evolution on the basis of their success at initiating local nucleation events early in the folding process [35]. Hecht and co-workers also found similar results when examining the binary patterning of polar and non-polar residues in proteins [38,39]. These authors observed that binary patterns with spacings of polar and non-polar residues matching the characteristic periodicity of particular secondary structures were more likely to be found in those secondary structures, leading them to conclude that the specific binary pattern of a protein sequence can influence the secondary structure which that sequence will adopt. Indeed, in an elegant empirical study that supports their statistical work, they showed that the binary patterning of residues has a greater effect on secondary structure determination than the secondary structural preferences of individual amino acids [40].

As one would expect, it is not just over-represented patterns that are of interest in these studies; those that are under-represented can be equally informative. For example, King and co-workers, in discussing the lower-than-expected number of hydrophobic runs from their analysis, note that it is more likely that these runs are suppressed due to constraints on the folding process rather than on structural constraints on native protein architecture [36]. Specifically, they note that, in concurrence with prior simulation results, hydrophobic runs may be selected against by evolution because they may promote off-pathway aggregation during folding [36,37]. Hecht and co-workers draw similar conclusions from the lower-than-expected frequencies of alternating polar-nonpolar patterns found in their work and others [39]. These alternating patterns might be thought to be favoured *a priori*, given that their periodicity matches that found in amphipathic *β*-strands. However, as patterns of alternating polar and nonpolar residues are known to promote amyloid-like structure in designed proteins, Hecht and co-workers suggest that alternating patterns are also subjected to negative selection by evolution to reduce amyloidogenesis during the folding process [38,40]. In both of these classes of studies, the statistically low results have been taking as an indication of *negative design*, a term referring to the need for protein sequences to not only code for the correct native structure, but also to code against competing structures. Schwartz and King, extrapolating from their statistical analyses, put it as follows: “amino acid sequence must be treated not merely as a “structural code” encoding a protein’s native state but also as a “folding code” providing additional information on how to get to and remain in the native state while avoiding pitfalls such as aggregation” [37].

### 2.3. Analyzing the Primary Sequence Using Information Theory

We conclude this section with a brief description of one further whole-sequence method that uses a different approach to sequence analysis: information theory. From an information point of view, protein folding can be seen as the transfer of information from one-dimensional chains of amino acids to three-dimensional folded proteins. In this context, Strait and Dewey [41], as well as Weiss *et al.* [42], have estimated the amount of information contained in protein sequences using Shannon information entropy [43]. Entropy, within information theory, is a widely-used measure expressed as the number of bits needed to store a set of data, and as such, it is inversely related to compressibility and redundancy. Random datasets, with minimal redundancy, have maximal entropy, and carry the maximum amount of information per unit of data. Weiss *et al.* note that it is very difficult to determine a reliable estimate of the sequence entropy because of the finite nature of the data been studied [42]. To circumvent this inherent limitation, they employ several different alphabet reductions to improve their statistics, from which they conclude that protein sequences are very nearly random, containing approximately 99% of the complexity of random sequences [42]. Strait and Dewey, on the other hand, drew different conclusions. Faced with the same problem of finite data, they used a variety of different techniques to corroborate their estimation that amino acids carry between 2.4 – 2.6 bits of information per amino acid, substantially less than the 4.2 – 4.3 bits of information carried by amino acids in random sequences [41]. These opposing results reflect another pitfall of statistical approaches: they may be based on underlying assumptions or have fundamental requirements that cannot easily be satisfied. In this case, the application of Shannon entropy analysis is compromised by the limited amount of data [42]. Moreover, this technique can produce varying results depending upon the extent of its application, and corroborating evidence may be needed to increase the reliability of the results. While information theory offers novel techniques for studying protein sequences, thus far it has been unable to unambiguously determine the degree of randomness in protein sequences.

## 3. Primary Sequence and Secondary Structure

### 3.1. Amino Acid Conformational Propensities

The statistical study of secondary structural sequence data began with the seminal study by Chou and Fasman that analyzed the residues within helices, *β*-structures and coil regions of 15 of the earliest available protein structures [44]. Despite the limited size of the dataset in this study, it was already evident that amino acids are not distributed randomly amongst the various types of secondary structure. These authors introduced the statistical term *conformational parameter* to describe the propensity for a residue to be found in a particular secondary structural environment. Conformational propensity values, which have come to be widely used throughout the literature, are normalized frequency values: the propensity for residue *r* in secondary structure *SS*, *P_r,SS_*, is given by:

(1) $$P_{r,SS}=[SS_{r}/SS_{all}]/[Total_{r}/Total_{all}]$$

where *SS_r_* and *SS_all_* are the number of *r* residues, and of all residues, in secondary structure *SS*, and *Total_r_* and *Total_all_* are the number of *r* residues, and of all residues, in the database. Numerous subsequent studies of secondary structure propensity values for *α*-helices [45–52], *β*-sheets [53–55], turns [56–59] and other secondary structural elements [60–63] have appeared in the literature, with general agreement. Ala, Glu, Met and Leu, for example, have high helix propensity values, while the short polar residues Ser, Thr and Asn, together with Gly and Pro, have low ones [51]. In contrast, these residues have approximately reversed propensities for turns [44].

Following the advent of additional structural data, more specialized statistical analyses became possible. Two groundbreaking papers published in the late 1980’s focused on residue positional preferences within helices. In one, Richardson and Richardson, using a set of 215 *α*-helices from 45 globular protein structures, took the notion of conformational propensities one step further and calculated position-specific residue propensities for a number of positions at both the N- and C-terminal (NT and CT) ends of helices [45]. As has been confirmed by numerous subsequent studies [46–48,50,64], short polar residues, along with Gly, are strongly favoured just prior to helices, while Pro, Glu and Asp are strongly favoured in the first few NT helical positions. Gly, on the other hand, is overwhelmingly favoured just after the last helical position, a location almost entirely devoid of Pro residues. At the same time, Presta and Rose, from a consideration of the incomplete hydrogen bonding patterns at the two ends of helices, proposed the helix hypothesis [65]. This hypothesis postulates that a necessary condition for helix formation is the presence of polar residues in the flanking regions around helices that can help to complete the missing hydrogen bonds at helical termini, thereby stabilizing nascent helices. Collectively, this research provides a prime example of how statistical results can foster empirical work: the discovery of *helix capping* – the sequential and structural motifs at the two helical termini that promote hydrogen bonding and hydrophobic interactions between the terminal helical residues and those immediately flanking the helical ends – has precipitated a large body of experimental research into the role of capping in helix stability [66–71]. Helix capping has also been included in both theoretical aspects of protein folding [72] and prediction methods [73]. In a broader sense, the idea of position-specific propensity values has been used to uncover residue preferences in other secondary structural environments, including at the termini of *β*-strands [60], in *β*- bulges [61], and within turns [56–59], among other environments [62].

As with studies of the primary sequence, a combination of statistical analyses of secondary structure and careful deduction can lead to inferences about the protein folding process itself. A clear example of this is found in two ingenious statistical studies of helices and turns by Chakrabarti and colleagues [50,59]. These authors examined the positional propensities of residues in the smallest helices (containing either three or four residues), and compared them with the positional propensities of residues in longer *α*-helices, and also with positional propensities of residues in *α*- and *β*-turns. The choice to examine only the smallest helices is noteworthy: as helical growth is thought to consist of an initial nucleation step followed by an elongation step, the authors hypothesized that the smallest helices have undergone nucleation only, and not subsequent growth. These short helices, therefore, may provide a means of identifying helix nucleation sites for subsequent analysis. Chakrabarti and colleagues [50] report a very high correlation between the residue propensities in the shortest helices and those found at the NT, but not the CT, end of longer *α*-helices, from which they draw two interesting implications for protein folding: *(i)* helix nucleation might occur predominantly at the N-terminus of helices; and consequently *(ii)* helical growth might be largely unidirectional, from NT → CT. Moreover, high correlations between the residues involved in the shortest helices and those found in both *α*- and *β*-turns led the authors to propose that *α*-helices might originate from *β*-turns, with either *α*-turns or short 3_10_-helices acting as intermediaries [50,59]. It is noteworthy that, though the NT of helices has been suggested by others to be involved in helix nucleation [74], these statistical studies have provided the only evidence to date in support of this proposition.

### 3.2. Residue Coupling

Residue coupling analysis investigates how residues pair together in various sequential relationships within secondary structures. A central hypothesis of this type of analysis is that the energetic effects of various types of pairing interactions will be reflected in a Boltzmann-like distribution of these pairings within a large, random sample of structures [75]. Thus, statistically elevated (reduced) pairing frequencies are interpreted to reflect favourable (unfavourable) underlying interactions [52]. In the case of helices, pairing preferences for various spacings have been tabulated, either along helices as a whole [27,51,76–80], or at helical termini [27,52]. The dominant pattern exhibited within helices reflects amphipathicity: the tendency for polar and non-polar amino acids to be segregated on opposite sides of a helix [76]. High coupling rates for pairs of hydrophobic residues, as well as pairs of hydrophilic residues, have been reported at *(i,i+3)* and *(i,i+4)* spacings within helices, precisely the spacings that bring residues next to one another spatially on a single helical face. Similarly, low coupling rates are reported at the *(i,i+2)* opposing-helical-face spacing. Pairings at the *(i,i+3)* and *(i,i+4)* spacings have received the lion’s share of attention, as these spacings capture the effects of side-chain–side-chain (SC-SC) interactions within helices [77–80]. Some studies use statistical coupling to test existing hypotheses about particular SC-SC interactions [77]. Most studies, however, perform what might be termed a statistical “fishing” exercise. Here, coupling analysis is performed on all possible pairings, and statistically significant ones are identified. Andrew *et al.*, for example, identified higher-than-expected pairing frequencies between polar and non-polar amino acids at the *(i,i+4)* same-helical face spacing, in contradiction to the dictates of amphipathicity [80]. The authors went on to verify this curious observation experimentally, thereby providing the first proof of polar-nonpolar stabilizing interactions in helices [80]. In some studies, a *lack* of correspondence between statistics and empirical observations was the dominant observation. Fernández-Recio and Sancho found poor correlation between pairing frequencies and measured SC-SC interaction energies in short helical peptides [79]. Their conclusions about this unexpected finding are noteworthy, and again demonstrate the type of inferences about protein folding that can be drawn from statistical work. Specifically, they conclude that the lack of correlation between SC-SC interaction energies and occurrence frequencies suggests that SC-SC interactions are poorly exploited by evolution for increasing helical stability [79]. They suggest that this may be because of: *(i)* an elevated role for tertiary interactions in helix stability, a proposition that lends support to folding models where secondary and tertiary folding are mutually stabilizing; and/or *(ii)* a requirement that proteins remain marginally stable for functional reasons, something that can be achieved if stabilization strategies are under-employed within protein structures. Indeed, despite clear amphipathic helical patterning, others have also questioned the importance of SC-SC interactions for helix stability [78].

Secondary structural coupling analysis has been used to study residue patterning in virtually all secondary structural environments. The residues in various turn positions, for example, have been examined for pairing preferences [59], as have the residues in helical capping positions [27,52] and loop regions [27,63]. Ordering within pairings has been explored, and pairing preferences have been found to be non-symmetrical [52]. But amongst the various secondary structural environments, it is *β*- architecture that has received the greatest attention from coupling analysis, most likely because so little is known about its folding process. Numerous studies have looked at coupling between both inter- and intra-strand pairs of residues within *β*-sheets in an effort to identify possible sequential motifs that might explain sheet origins [27,53–55,81–84]. Early studies showed that inter-strand *β*-sheet pairings favoured electrostatic and hydrophobic compatibility [81], though in a non-specific manner. Lifson later showed that inter-strand pairing preferences were actually *specific*: Ser/Thr, Ile/Val, Ile/Leu and (Lys,Arg)/(Asp,Gln) were all found to have higher-than-expected pairing frequencies between strands [82]. Pairs of Cys residues have also been reported to occur more often than expected in neighbouring cross-strand positions [54,84]. Moreover, the complexities of *β*-architecture have allowed for more specific coupling analyses: inter-strand pairing preferences have been calculated separately for parallel and anti-parallel strands [27,54,83,84], for edge and interior strands [27], and for hydrogen bonded and non-bonded positions within strands [27,54,83,84]. Following from these statistical studies, the conformations adopted by interacting, cross-strand side-chains have been investigated [54,83,84]. Characteristic conformations for specific residue pairings are commonly seen, each with intimate SC-SC contacts between the residues that often involve hydrogen bonding and nesting along the length of the side-chain that promotes van der Waals interactions. Interestingly, a number of coupling studies of *intra*-strand pairings did not find any statistically significant variations from random in *(i,i+2)* residue pairings [81,83], a somewhat unexpected finding given that this spacing will place two residues spatially next to one other on the same side of a *β*-sheet. The distance between these pairings is generally thought to be too great to allow for such *(i,i+2)* SC-SC interactions [81,83].

As with other types of statistical analyses, the examination of residue coupling within secondary structures has lead to general inferences about the process of protein folding. In a frequently cited study, for example, Curmi *et al.* found statistical evidence suggesting that electrostatic compatibility was a key component in the pairing preferences of residues on adjacent *β*-strands [54]. However, they did not observe any particular electrostatic SC-SC interactions between strands within protein structures that would account for the elevated electrostatic pairing frequencies. The authors solve this potential disparity in their findings by moving beyond protein structure: they propose that the elevated electrostatic pairing frequencies do not reflect contributions to structural stability of *β*-sheets, but rather contributions to the process of *β*-structure formation itself. Specifically, they posit that electrostatic interactions may be critical for establishing the correct strand-strand register, or alignment, early in sheet development [54]. Such initial aligning SC-SC interactions, they contend, may not necessarily carry forward to final protein tertiary structure. In another study, Mandel-Gutfreund *et al.* draw contrasting conclusions about sheet formation through the use of a different statistical approach: conservation and covariance [85]. In this case, the authors do not analyze pairing preferences in a non-redundant dataset, but instead look at how individual amino acids in pairs of neighbouring residues on adjacent strands have mutated within families of evolutionarily related proteins. They report that, while buried pairs are more conserved than those on protein surfaces, the degree of covariance and conservation observed between neighbouring residues on adjacent strands, regardless of whether they are buried or not, is not statistically different than that observed between other, non-neighbouring pairings [85]. From this counter-intuitive observation, the authors conclude that the evolutionary pressure for residue complementarity between pairs on adjacent strands is weak [85]. Sheet formation, they suggest, is not as dependent on specific SC-SC interactions after all.

## 4. Primary Sequence and Tertiary Structure

It is widely recognized that the relationship between a protein’s primary sequence and its tertiary structure is based on a combination of both secondary and tertiary interactions [86], and as such, no simple connection between the two is expected. Overarching rules – if they exist – are extraordinarily elusive. A number of inferences about protein folding from studies of the primary sequence alone and in relation to secondary structure have been presented in previous sections. Here, we conclude this survey of the statistical study of protein sequences by examining how sequence analysis has been used within three important fields of tertiary structure research: separating structured from unstructured proteins, identifying domain boundaries within structured proteins, and predicting the structural classes of protein domains.

### 4.1. Natively Disordered Proteins

There is now abundant evidence that a substantial portion of proteins, particularly in eukaryotes, lack definitive structure under physiological conditions, yet these disordered, or natively unfolded, proteins are increasingly found to have important functional roles within cells [87–89]. The discovery of functional proteins that lack stable, tertiary structure also adds a level of complexity to the sequence-structure relationship, as it divides the population of sequences into two: those that yield natively ordered, and those that yield natively disordered, proteins. This development serves as a reminder that the protein structures solved to date – and therefore the statistics that have been derived from them – do not necessarily reflect the characteristics of all proteins, but rather are biased towards those proteins that are amenable to structure determination, namely those that can be cloned, expressed, purified and solved by X-ray crystallization or NMR. Moreover, this novel class of proteins can also serve to remind about the importance of flexibility for protein architecture and interactions, something that can easily be lost when considering the more rigid concept of protein ‘structure.’ Because of the clear structural and functional differences between these two classes of proteins, it is natural to ask whether there are any sequential differences between them. As in other areas of protein folding studies, statistical analysis has been employed to address this question. Uversky *et al.*, for example, have used comparative statistics to search for differences in the sequences of these two populations [90]. While characteristics such as polypeptide length, net charge and pI were not found to differentiate between natively folded and unfolded proteins, these authors demonstrated that a combination of mean net charge and mean hydrophobicity can be used to distinguish between them. The authors further suggest that natively unfolded proteins can be induced to adopt folded conformations through interactions with other proteins or ligands that produce an increase in the mean hydrophobicity and a decrease in the mean net charge in the resulting complex [90]. This work therefore supports a model of protein folding that is based on the creation of a stabilizing hydrophobic core built from residues located, not in isolated segments of a chain, but throughout the entire chain length. In addition, others have shown that disordered proteins have lower sequence complexities, as measured using information theory [91], and have differences in their amino acid composition [91,92], than are found in natively ordered proteins. Thus, while this is a relatively new field of study within the larger context of protein folding, the importance of statistical analysis to this field is already evident.

### 4.2. Domain Boundary Prediction

A related area of study within protein folding is domain boundary prediction. Domains are autonomously folding units within proteins [93,94] that are separated from one another by linker regions. These linker regions are often disordered, and are thus structurally similar to natively unfolded proteins. While most techniques for identifying domain boundaries make use of evolutionary information [94,95] or machine learning techniques [96–99], there have been several statistical attempts in recent years to identify domain boundaries by direct examination of a protein’s amino acid sequence [100–102]. Most have combined structural insights with relatively simple statistical measures to achieve reasonable success at boundary prediction. For example, Wheelan *et al.*, noting the narrow range in the number of residues observed in domains of solved proteins, achieved surprisingly successful boundary prediction by making what amounts to educated guesses, using the statistical distribution of domain lengths to guide their predictions [100]. Galzitskaya and Melnik, on the other hand, used a statistical approach to domain boundary prediction that was grounded on an interesting premise: they hypothesized that, for protein folding to occur, the amount of entropy lost along a protein chain during folding (approximated for each residue by the number of dihedral angles in its backbone and side-chain) must be compensated for by localized enthalpy gains [102]. Given this, the authors conclude that those parts of a chain with high potential for entropy loss will be the primary loci of folded domains, as the domain architecture will provide the close packing in these regions required to offset their high entropy losses. Using this, they built a predictive scheme that statistically scored the entropy within a sliding window of residues, and predicted domain boundaries at entropic minimums [102]. Finally, other methods have based their predictions on statistical differences in amino acid composition between linker regions and folding domains [101], with some success.

### 4.3. Domain Structural Class Prediction

Domain structure is another area of protein research that has received considerable attention. At the atomic level, no two domain structures are the same, but looked at from a topologically higher level, structural patterns begin to emerge. An early study by Levitt and Chothia [103] found that domains could be separated into four structural classes: all-*α*, all-*β*, *α*+*β*, *α*/*β*. This basic classification is still widely used today. For a protein with unknown structure, knowledge of its structural class is of importance for numerous reasons [104], not the least of which is that this information can greatly improve the accuracy of structure prediction. Consequently, structure class prediction has been an active area of research for many years, with statistical approaches used extensively [104–109]. An early study by Nakashima *et al.* analyzing the amino acid composition of proteins in different structural classes showed that proteins tended to cluster into their own regions of 20-dimensional space, showing directly that a protein’s amino acid composition (AAC) is correlated to its structural classification [105]. This significant finding led to the development of a number of prediction techniques based on the statistical differences in AAC across structural classes [104,106,107]. Chou and Zhang [104] improved the predictive power of AAC methods by introducing a new distance metric into the analysis, the Mahalanobis distance [110], which included coupling, or statistical covariance, between amino acids. Others expanded on the basic approach by replacing AACs with distributions of physiochemical properties of the amino acids [106]. Hybrid approaches have also been reported [107]. In general, all of these methods exploit the statistical relationship between the amino acid composition of a protein and the structural fold of that protein, though the specific nature of this relationship remains unknown. Other groups have used simpler statistical methods to achieve considerable predictive success, relying on differences in the occurrence frequencies of di-, tri- and quad-peptides within the different structural classes for making predictions [108,109].

## 5. A Novel Example: Residue Patterning in *β*-Strands

We conclude this introduction to the statistical study of residue patterning within protein sequences by reporting novel findings from our lab regarding residue patterning within *β*-strands. To better illustrate how the use of statistics can aid in investigating problems associated with protein folding, we first briefly describe the motivations of this present work.

It has long been postulated that proteins undergo a transition from the unfolded to the folded state as a result of hydrophobic collapse [6,29,30]. Virtually all globular proteins fold so that the majority of their hydrophobic residues become buried in their core, thus reducing their exposure to water [111]. Although the specific mechanism(s) leading to the formation of *β*-structures are not known [112], this general principle of hydrophobic sequestration has also been proposed to account for the formation of *β*-sheets [6,113]: hydrophobic residues on separate strands make initial contacts to form hydrophobic clusters, and sheet formation ensues around these clusters. This model requires specificity, both in terms of a non-random distribution of key hydrophobic residues in and around *β*-strands, and in terms of the residues surrounding these key hydrophobic residues. Without such specificity, there will not be a way for native, on-pathway hydrophobic contacts to distinguish themselves from non-native, off-pathway ones. Thus, under this folding model the local patterning of hydrophobic residues in and around *β*-strands is critical for achieving correct native folds.

Given this model for *β*-sheet nucleation that involves both hydrophobicity and residue specificity, we hypothesize that the distribution of hydrophobic residues within *β*-strands is not random, but rather must contain local aggregations that provide the specificity necessary for correct strand-strand recognition during *β*-sheet nucleation. We are therefore chiefly interested to see if there is an increased frequency of *(i,i+2)* same-sheet-surface hydrophobic pairings on *β*-strands. As outline in previous sections, numerous studies of hydrophobic patterning within *β*-sheets and within protein sequences generally have appeared in the literature [28,35–40,54,55,81–84]. Some find that protein sequences are essentially random [28], while others do not [35–40]. Specifically with regards to *β*-strands, two studies have reported that the frequency of *(i,i+2)* pairing cannot be distinguished from random [81,83], contrary to our hypothesis. To investigate this further, we have re-examined the coupling of hydrophobic residues in *β*-strands anew, this time extending the analysis of previous works by looking at position-specific residue coupling.

### 5.1. Methods

For our investigation, we compiled a non-redundant dataset of protein structures using the PDB-REPRDB database [114], which consists of representative protein chains from the RCSB Protein Data Bank, release #2007_01_27. Only protein chains solved by X-ray crystallography with resolutions less or equal to 2.0 Å, and R-factors less than or equal to 0.25 were considered. In addition, to be included, protein chains had to be free of chain breaks, have coordinates for all non-hydrogen atoms, and be at least 40 residues in length. Membrane proteins, as well as those polypeptide chains that were part of larger structural complexes, were discarded. To ensure non-redundancy in our dataset, only one representative was chosen from amongst those protein chains that had sequence identities greater than 30%, or structural alignments less than 10 Å. In all, our dataset consisted of 1338 protein chains. *β*-strands were identified in our non-redundant dataset using the program DSSP [115]. A total of 11,682 strands of length 3 or more were identified and used in our study, of which 3565 were parallel and 7023 were anti-parallel.

With this dataset, we calculated coupling statistics for pairs of hydrophobic residues (VLIFM), and pairs of hydrophilic residues (KREDSTNQ), at various spacings in *β*-strands. Expected values and standard deviations were calculated using a shuffling procedure as follows. For each query pattern, the actual frequency was first tabulated within our dataset. Following this, the amino acids within all strands *of sufficient length* (*ie*. all strands at least as long as the query pattern) were redistributed randomly 1,000 times, and a new count of the query pattern frequency was made after each randomization. The resulting 1,000 counts were used to determine an expected value and a standard deviation for the query pattern, providing a means of evaluating the actual frequency count. Statistics were calculated separately for *(i)* all strands, *(ii)* parallel strands, and *(iii)* anti-parallel strands.

### 5.2. Results and Discussion

**Figure 1.**
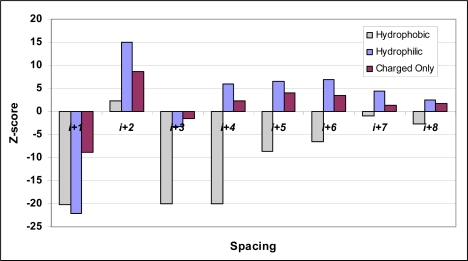
Hydrophobic and hydrophilic coupling within *β*-strands.

Using *z*-scores to compare measured and expected frequencies, we find that both hydrophobic and hydrophilic pairings occur with non-random frequencies in *β*-strands (Table 1, Figure 1). Hydrophilic pairs exhibit clear amphipathic patterning at the *(i,i+1)* through *(i,i+4)* spacings, while at further spacings these pairings occur more often than expected, though the statistical significance tapers towards randomness at larger spacings. The same trends hold for pairs of charged residues (KRED) and neutral hydrophilic residues (STNQ), calculated separately (data not shown). Hydrophobic pairs, in contrast, are found with low frequencies at virtually all spacings, implying that the distribution of hydrophobic residues within *β*-strands does not reflect amphipathicity. Pairs of hydrophobics are particularly suppressed at *(i,i+1)*, *(i,i+3)* and *(i,i+4)* spacings, with calculated *z*-scores of −20.3, −20.0 and −20.1 respectively. The values for the *(i,i+1)* and *(i,i+3)* spacings suggest that hydrophobics are strongly discouraged from being on opposite sides of *β*-sheets, consistent with amphipathicity, but the marginally elevated *(i,i+2)* value (*z*-score 2.2) and the highly suppressed *(i,i+4)* do not suggest that hydrophobic residues are encouraged to be near one another on the same *β*-surface.

**Figure 2.**
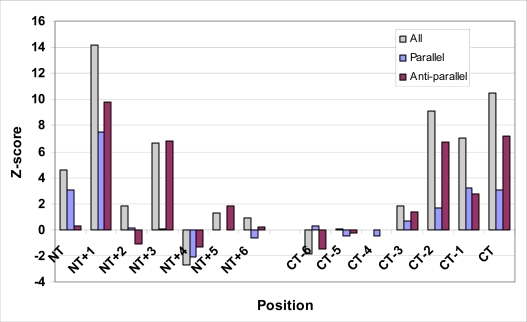
Position-specific hydrophobic coupling within *β*-strands.

These results imply that residue distributions in *β*-architecture are non-random. However, at the critical *(i,i+2)* spacing that is the focus of this investigation, there is less certainty. Hydrophilic residue pairs occur at this spacing with frequencies much higher than would be expected if the hydrophilic distribution was random; however, the frequency of hydrophobic pairings is much closer to what would be expected for a random hydrophobic distribution. This concurs with an early study by Heijne and Blomberg [81], as well as a more recent one by Hutchinson *et al.* [83], who found no evidence of *(i,i+2)* hydrophobic coupling. To investigate this further, we extended our coupling analysis by examining position-specific coupling at various positions relative to the NT and CT ends of strands. Though other types of secondary structure have been so studied, to our knowledge position-specific coupling analysis has not been applied to *β*-architecture. Table 2 and Figure 2 contain the position-specific *(i,i+2)* pairing frequencies for hydrophobic residues at positions ranging from *(NT,NT+2)* through *(NT+6,NT+8)*, as well as for positions ranging from *(CT-8,CT-6)* through *(CT-2,CT)*. From this figure, it is evident that the frequency of hydrophobic *(i,i+2)* pairings is not uniform across all positions. Instead, there are high levels of hydrophobic coupling at the two termini, particularly at the *(NT+1,NT+3)* position, and at the *(CT-4,CT-2)* and *(CT-2,CT)* positions. In contrast, at central positions the coupling frequency cannot be distinguished from what would be expected from a random hydrophobic distribution. Moreover, the frequencies of hydrophobic *(i,i+2)* pairings differ markedly between parallel and anti-parallel strands. On parallel strands, *(i,i+2)* pairings of hydrophobic residues occur with high frequency at the *(NT+1,NT+3)* position, and show only marginally increased frequencies at the CT. On the other hand, hydrophobic *(i,i+2)* pairs are found with high frequencies on anti-parallel strands in tandem positions at both the NT and CT: positions *(NT+1,NT+3)*, *(NT+3,NT+5)*, *(CT-4,CT-2)* and *(CT-2,CT)* all have high hydrophobic pairing frequencies. In short, parallel strands have increased hydrophobic pairing only at their NT, while anti-parallel pairs have increased hydrophobic pairing at both of their termini. Interestingly, if one considers the orientations of strands, the increased hydrophobic pairings at both ends of anti-parallel strands may simply reflect hydrophobic patches at one end of anti-parallel strand *pairs*, a conjecture that is supported by the curious finding of increased hydrophobic pairings at tandem positions of both anti-parallel termini. Under this interpretation, pairs of both parallel and anti-parallel strands can be seen to have increased hydrophobic pairing at one of their ends.

We therefore find evidence of increased hydrophobic *(i,i+2)* pairings in *β*-strands. These results differ from those of early studies [81,83], but this difference can be attributed to the uneven distribution of such pairings on strands: there are specific locations at strand termini that show strong *(i,i+2)* hydrophobic coupling, while frequencies at other locations suggest that no such coupling exists.

Of note about this and other statistical techniques that use a large, non-redundant dataset of protein structures to investigate the sequence/structure relationship is that the findings should be independent of the structural peculiarities of any of the specific proteins in the dataset. Thus, in this case, our findings reflect the characteristics of an idealized *β*-strand, created from an average over all the strands in our dataset. The effects of interesting structural phenomena, such as *β*-bulges which would reverse the strand sense, will not detract from the major coupling patterns found on *β*-strands, providing the occurrence of unusual structural phenomena such as *β*-bulges is randomly distributed along strands.

As with any statistical result, there is always the question of interpretation. In this case, we interpret our results to support a model of sheet nucleation that involves hydrophobic interactions between disparate parts of the protein chain. As mentioned, such a model requires specificity: non-native sheet nucleation can impair protein folding and lead to aggregation problems such as is seen in amyloidogenesis [116]. Indeed, others have speculated that the low frequency of *(i,i+2)* hydrophobic pairings across protein sequences reflects the principle of negative design in evolution [38,40] which seeks to minimize inadvertent sheet formation. In this light, it is not surprising to find that *β* -sheets do not have an elevated frequency of *(i,i+2)* hydrophobic pairs. However, as native sheets also require nucleation, a localized increase in *(i,i+2)* hydrophobic pairings can still be expected. Our position-specific analysis finds precisely this: increased hydrophobic pairings at strand termini. One possible explanation for our findings, therefore, is that the sites of sheet nucleation are located predominantly at the edges of sheets. A corollary to this is that, following nucleation between two disparate parts of the chain, the majority of *β* growth between these chains is unidirectional. For parallel strands, this direction can be specified as NT → CT. Moreover, it is curious that the increased *(i,i+2)* hydrophobic coupling occurs at the *(NT+1,NT+3)* position, and not the *(NT,NT+2)* position. It may be that the NT position directly before a nucleation seed is itself involved in the seed (thereby conferring additional specificity) in such a way as to block subsequent growth towards the chain NT.

While this interpretation is consistent with our hypothesis, it must be remembered that statistics alone cannot provide physical explanations. Lifson noted as such many years ago: “[t]he physical reasons for the particularly favourable or unfavourable pairs are impossible to determine by statistical analysis” [82]. Another interpretation of our results, for example, can be traced back to an early study of sheet architecture by Cohen *et al.* These authors, when studying *β*-sheet sandwich architecture, noted that the major hydrophobic interactions between sheets occur at the sheet corners [117]. Although their study is limited to a single class of *β*-architecture, it may be that this is a wider phenomenon, and that the increased hydrophobic pairing that we find at strand termini is a reflection of such a phenomenon. We are presently extending our work beyond single strands to investigate this possibility, as well as the nature of the hydrophobic distribution across sheets in general.

## 6. Concluding Remarks

We have attempted to give a general introduction into the use of statistics for studying residue patterning within protein sequences. As this is an extended topic, we have aimed at breadth rather than depth, though even covering the full breadth of statistical pattern mining in protein chains is a very large undertaking. Consequently, we have chosen those areas of research that we feel best exemplify the power of statistical analyses for drawing inferences about events that are difficult or even impossible to study empirically, such as early events in protein folding or details about the evolutionary origins of modern-day proteins.

Statistical analyses provide a powerful way of summarizing properties of protein sequences that can easily go unnoticed, particularly when attention is focused on a single or a small number of protein structures. As such, a broad survey of protein sequence and structure may uncover connections that can then be investigated experimentally. Part of their utility comes from their ease of use: it is almost always far easier to analyze a set of protein sequences then devise and implement a series of experiments to study a particular phenomenon.

Despite its utility, there are important limits to what can be accomplished with statistics. The interpretation of results is an important part of research, but it must be remembered that such efforts are solely inferences. Cause and effect are often difficult to untangle. We conclude by revisiting the words of Lifson and Sander, which aptly describe the limits of statistical analysis:

“… the limitations in all statistical approaches to protein structure must be kept in mind: proteins are biologically evolved molecules with a particular structure tailored to a particular function. Only average properties of protein structures can be understood by statistical methods, but not the highly individual character of each protein” [53].

## Figures and Tables

**Table 1.** Coupling analysis between pairs of hydrophobic (VLIFM), and pairs of hydrophilic (KREDSNTQ), residues at various spacings within *β*-strands.

Spacing	Hydrophobic			Hydrophilic		
	Frequency	Expected	Z-score	Frequency	Expected	Z-score
*i+1*	9361	10592.9	−20.3	4579	5751.3	−22.2
*i+2*	8291	8161.8	2.2	5467	4716.4	15.1
*i+3*	4803	5787.4	−20.0	3475	3614.1	−3.2
*i+4*	2871	3759.1	−20.1	2950	2714.0	5.9
*i+5*	1960	2258.3	−8.6	2197	1983.0	6.5
*i+6*	1133	1322.7	−6.6	1526	1339.4	6.8
*i+7*	738	758.4	−1.0	988	889.8	4.3
*i+8*	386	428.4	−2.6	615	571.8	2.5

**Table 2.** Position-specific coupling analysis between pairs of hydrophobic (VLIFM) residues across all strands, parallel strands, and antiparallel strands.

Pairing	All Strands			Parallel Strands			Antiparallel Strands		
	Freq.	Expect	Z-score	Freq.	Expect	Z-score	Freq.	Expect	Z-score
NT,NT+2	1976	1835.1	4.6	802	748.1	3.0	952	944.9	0.3
NT+1,NT+2	2450	1937.3	14.2	1004	853.4	7.5	1181	948.7	9.8
NT+2,NT+4	1463	1408.7	1.8	522	519.8	0.1	718	743.6	−1.1
NT+3,NT+5	1086	916.2	6.6	244	243.2	0.1	685	551.9	6.8
NT+4,NT+6	498	550.7	−2.7	81	96.9	−2.1	348	370.4	−1.3
NT+5,NT+7	345	324.8	1.3	31	31.2	0.0	264	239.1	1.8
NT+6,NT+8	191	180.8	0.9	11	13.0	−0.6	137	134.5	0.2
CT-8,CT-6	168	189.7	−1.9	14	13.1	0.3	127	142.1	−1.4
CT-7,CT-5	339	338.0	0.1	32	34.2	−0.5	245	248.4	−0.3
CT-6,CT-4	571	571.1	0.0	102	105.8	−0.5	379	379.0	0.0
CT-5,CT-3	995	947.5	1.9	268	259.7	0.7	593	565.3	1.4
CT-4,CT-2	1722	1439.1	9.1	568	539.4	1.7	910	753.7	6.7
CT-3,CT-1	2204	1958.5	7.0	925	859.1	3.2	1029	961.3	2.7
CT-2,CT	2032	1719.0	10.5	789	735.6	3.0	1021	857.9	7.2
